# Supplementary figures and images for: The Telomeric Repeats of HHV-6A Do Not Determine the Chromosome into Which the Virus Is Integrated
Source: Genes (Basel). 2023 Feb 18;14(2):521. doi: 10.3390/genes14020521 (PMC9957103; doi:10.3390/genes14020521)

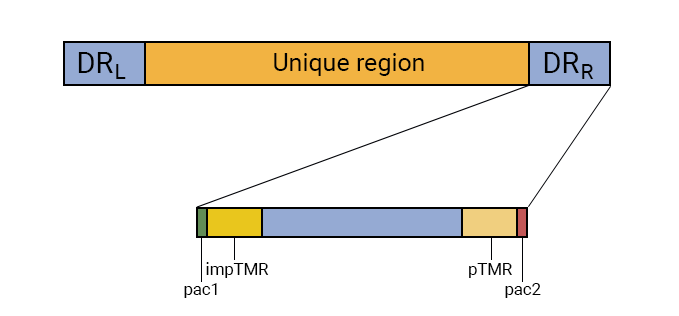

Supplement: Supplementary file 1 [file genes-14-00521-s001.zip › Fig1.png]

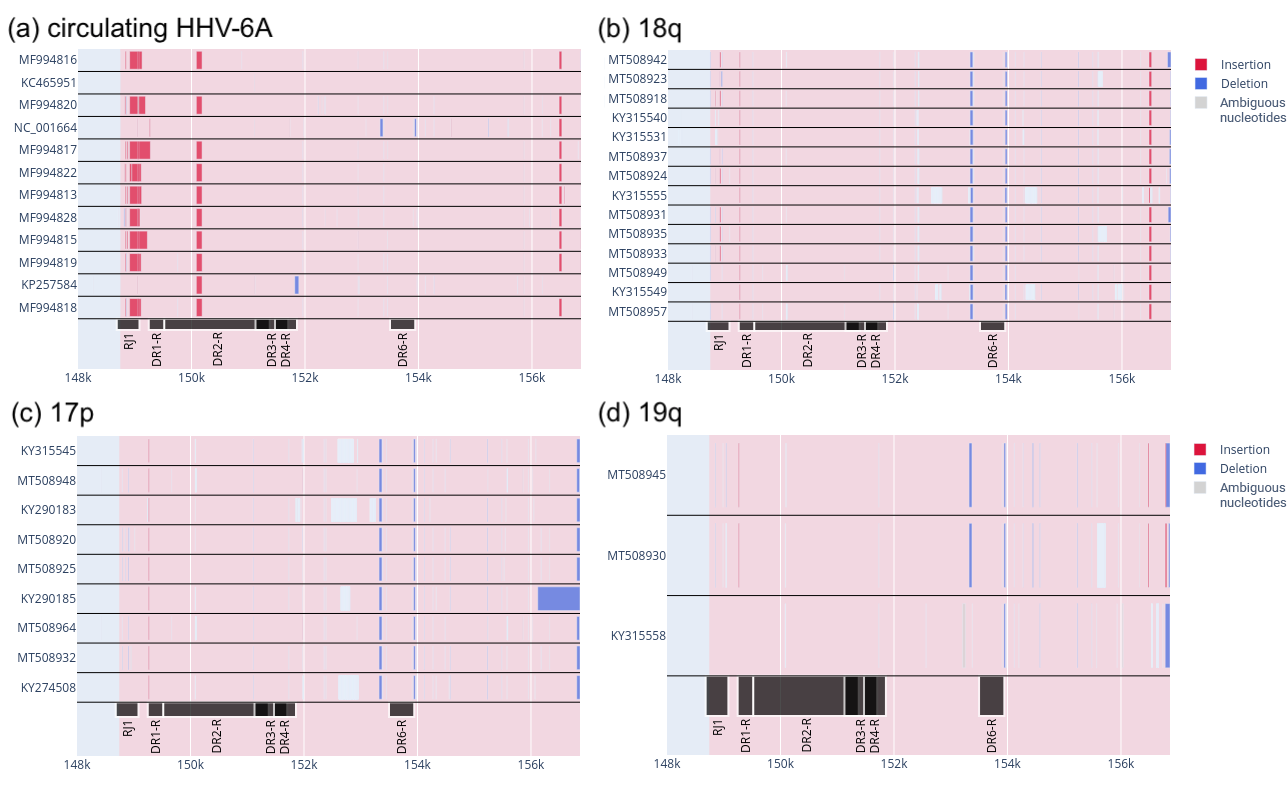

Supplement: Supplementary file 1 [file genes-14-00521-s001.zip › Fig2.png]
